# Can we consider discontinuation of hypomethylating agents in patients with myelodysplastic syndrome : a retrospective study from The Korean Society of Hematology AML/MDS Working Party

**DOI:** 10.18632/oncotarget.18258

**Published:** 2017-05-29

**Authors:** Da Jung Kim, Ho Sup Lee, Joon-Ho Moon, Sang Kyun Sohn, Hyeoung Joon Kim, June-Won Cheong, Deog-Yeon Jo, Hawk Kim, Hyewon Lee, Soo-Mee Bang, Won Sik Lee, Yong Park, Mark Hong Lee, Jae Hoon Lee, Sung Hwa Bae, Min Kyoung Kim, The Korean Society of Hematology AML/MDS Working Party

**Affiliations:** ^1^ Department of Internal Medicine, Kosin University College of Medicine, Kosin University Gospel Hospital, Busan, South Korea; ^2^ Department of Hematology/Oncology, Kyungpook National University Hospital, Daegu, South Korea; ^3^ Department of Hematology-Oncology, Chonnam National University Hwasun Hospital, Hwasun, Jeollanamdo, South Korea; ^4^ Department of Internal Medicine, Yonsei University College of Medicine, Seoul, South Korea; ^5^ Department of Internal Medicine, Chungnam National University Hospital, Chungnam National University, Daejeon, South Korea; ^6^ Division of Hematology and Cellular Therapy, Ulsan University Hospital, University of Ulsan College of Medicine, Ulsan, South Korea; ^7^ Department of Hematology-Oncology, Center for Hematologic Malignancy, National Cancer Center, Goyang, South Korea; ^8^ Department of Internal Medicine, Seoul National University Bundang Hospital, Seoul, South Korea; ^9^ Department of Internal Medicine, Busan Paik Hospital, Busan, South Korea; ^10^ Department of Internal Medicine, Korea University College of Medicine, Seoul, South Korea; ^11^ Division of Hematology-Oncology, Department of Internal Medicine, Konkuk University Medical Center, Konkuk University School of Medicine, Seoul, South Korea; ^12^ Department of Internal Medicine, Gachon University Gil Medical Center, Incheon, South Korea; ^13^ Department of Internal Medicine, Daegu Catholic University Medical Center, Daegu, South Korea; ^14^ Department of Hematology-Oncology, Yeungnam University Medical Center, Yeungnam University School of Medicine, Daegu, South Korea

**Keywords:** myelodysplastic syndrome, discontinuation, survival, decitabine, azacitidine

## Abstract

It is often difficult to continue treatment with hypomethylating agent(HMA) in clinical practice because of problems such as toxicities, poor economics, etc. We compared clinical outcomes of those patients who continued HMA and those who discontinued HMA because of other causes, and evaluated factors associated with survival in those patients who discontinued HMA.

Patients were divided into two groups: treatment failure, those who stopped treatment due to disease progression; and discontinuation, those who discontinued treatment because of other causes.

The median progression free survival(PFS) was 9.2 months (range 7.7 – 10.7 months) vs 28.9 months (range 22.6 – 35.2) in the treatment failure and discontinuation groups, respectively (*P* < 0.001). In a multivariate analysis, a lower risk by WPSS was an independent predictive factor for a longer PFS, and a lower risk by WPSS and median number of HMA cycles greater than seven were independent predictive factors for longer overall survival(OS) only in the discontinuation group.

Patients who discontinued HMA without disease progression showed a prolonged survival than those who failed HMA treatment. Especially, a lower risk by WPSS and longer duration of HMA treatment may be predictive factors for a longer PFS and OS in patients who discontinued HMA.

## INTRODUCTION

Myelodysplastic syndromes (MDS) are a heterogeneous collection of clonal hematopoietic malignancies that primarily affect the elderly and are characterized by bone marrow failure and dysplasia [1]. They are characterized by poor overall survival (OS) due to ineffective hematopoiesis, progressive cytopenias, and transformation to acute myeloid leukemia (AML). The hypomethylating agents (HMAs), azacitidine and decitabine, are commonly and effectively used to treat MDS; about half of high risk patients who were not eligible for allogeneic stem cell transplantation had hematologic improvement and a survival benefit with azacitidine for about 2 years [2–7]. Although treatment with HMA may prolong survival in patients with a poor prognosis, there are still a significant proportion of patients with MDS who do not respond to therapy with HMA and patients who lose response or progress on therapy [8–10]. According to previous studies, prolonged treatment duration may contribute to survival benefit [4, 11]. The optimal duration of treatment is unknown, but continued therapy for as long as a response is maintained is generally recommended [12]. Continuation of therapy is particularly important in patients who achieve a complete response (CR). For those patients who stop HMA treatment, most lose any response [13].

There are known prognostic factors for survival in MDS. The Global M.D. Anderson Scoring System (MDGSS) risk model, which includes poor performance, older age, thrombocytopenia, anemia, increased bone marrow blasts, leukocytosis, chromosome 7 or complex (≥ 3) abnormalities, and prior transfusions, allows risk assessment of any patient, regardless of prior therapy, at different time points in the course of the disease [14]. It was found to be independently predictive of outcome in patients with higher-risk as well as lower-risk MDS [15, 16]. Age, bone marrow blast count, and cytogenetics were found to have prognostic value in lower or higher risk patients who were treated with HMA [17, 18].

However, in clinical practice, though not in clinical trials, it is difficult to continue treatment of HMA because of problems such as toxicity, poor economics, comorbidities, compliance issues, etc. For those reasons, discontinuation of HMA before disease progression often happens in the clinical setting. That said, sometimes patients who stop treatment without progression show a long term survival. Cabrero et al. suggested that those patients who received more than 12 courses of HMA therapy and did not have high-risk cytogenetics had a significantly longer OS and tended to also have longer progression-free survival (PFS) [19]. On the other hand, there is a poor outcome in patients with lower-risk MDS who fail HMA treatment (median survival, 17 months) [15]. So, there are some questions about survival in MDS patients treated with HMA in clinical situations. Are there any differences of clinical outcomes between patients who continued HMA until disease progression and those who discontinued HMA because of other causes? And which factors will be helpful to predict survival in patients who discontinued HMA without disease progression? The purpose of this study was to look for answers to the above questions.

## RESULTS

### Patient characteristics

**Figure 1:**
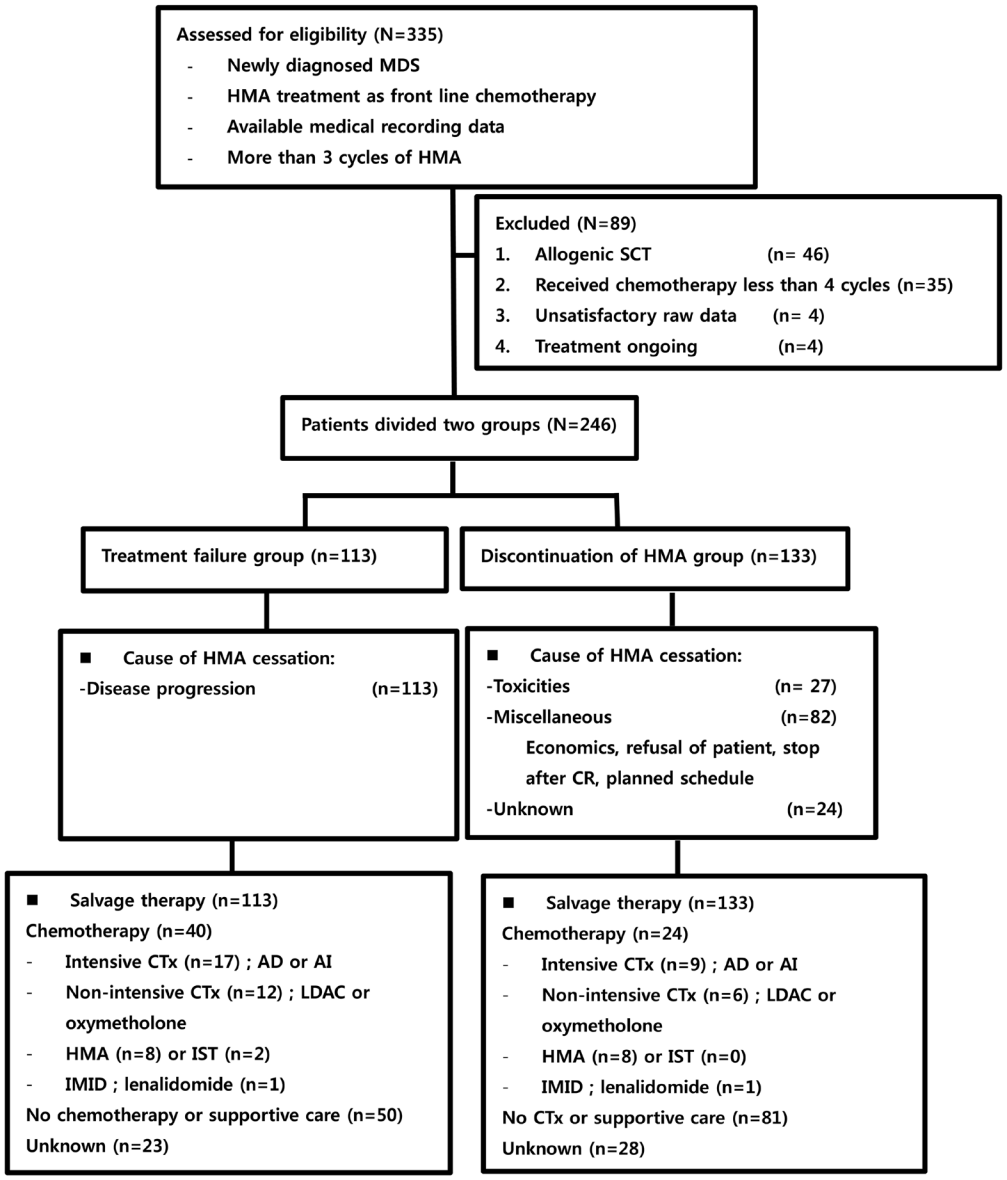
Study selection and flow chart MDS: myelodysplastic syndrome; HMA: hypomethylating agents; CR: complete response; CTx: chemotherapy; AD: cytarabine plus daunorubicin; AI: cytarabine plus idarubicin; LDAC: low dose cytarabine; IST: immune suppressive therapy; IMID: immune modulatory drug.

From January 2001 through October 2013, a total of 335 patients were collected in this study and 246 patients were analyzed. All estimated patients were diagnosed with MDS and received HMA as first line treatment. Among the excluded eighty nine patients, 46 received allogenic stem cell transplantation, 35 received less than 4 cycles of HMA, 4 did not have satisfactory raw medical records, and 4 were receiving ongoing HMA treatment. One hundred thirteen patients stopped HMA therapy because of disease progression, but 133 patients stopped HMA therapy because of other causes, such as toxicities, costs, patient refusal, planned schedule, etc. Twenty seven patients suffered from toxicities of chemotherapy, 82 refused treatment because of economics or personal reasons, and some patients were to receive 12 months of HMA chemotherapy as directed by their physicians. Additionally, 24 patients were lost to follow-up. After confirmed progression or relapse, salvage therapies were applied to patients. Chemotherapy was given to forty patients in the treatment failure group and 24 in the discontinuation group. No treatment or supportive care was administered in fifty and eighty one patients in both group, respectively. Further, information regarding salvage therapy could not be found in twenty three and twenty eight patients in the two groups, respectively. Detailed salvage therapy is presented in Figure 1.

The median age of the patients was 68 years (range 24-92 years) and the male to female ratio was 1.9:1.0. One hundred seventy five patients were treated with azacitidine (71.1%) and 71 patients with decitabine (28.9%). The median number of HMA cycles was 7 (range 4-63 cycles). All patients were stratified by IPSS, WPSS, and IPSS-R risk models.

### Comparison of treatment outcomes between the two groups

The comparison of characteristics between the treatment failure and discontinuation groups is shown in Table 1. The percentage of patients with lower than 5% bone marrow myeloblasts was 37.9% in the treatment failure group and 62.1% in the discontinuation group (*P* = 0.015). Patients who were at lower risk by IPSS or WPSS were documented in the discontinuation group more than in the treatment failure group. The treatment failure and discontinuation groups included 60 and 87, or 41 and 72 low risk patients, depending on the use of IPSS or WPSS, respectively (*P* = 0.050 and 0.005). However, patients included in the treatment failure group received salvage chemotherapy more than those in the discontinuation group (62.5% vs 37.5%, *P* = 0.006). The best responses were documented in all patients. 41.5% of patients were assessed with PR or more than PR, and 44.3% of patients had stable disease (SD) or only HI. The median PFS was 14.2 months (range 11.9-16.5) and the median OS was 30.7 months (range 25.3-36.1) in all patients.

**Table 1:** Comparison of characteristics between treatment failure and discontinuation of HMA group

Value (%)	Treatment failure group (n=113)	Discontinuation of HMA group (n=133)	*P*-value
**Age, years**			0.283
** < 65**	40 (41.7)	56 (58.3)	
** ≥ 65**	73 (48.7)	77 (51.3)	
**Sex (%)**			0.894
** Male**	73 (45.6)	87 (54.4)	
** Female**	40 (46.5)	46 (53.5)	
**ECOG**			0.434
** < 2**	97 (46.0)	114 (54.0)	
** ≥ 2**	10 (55.6)	8 (4.4)	
**BM blast, %**			0.015
** < 5**	44 (37.9)	72 (62.1)	
** ≥ 5**	67 (53.6)	58 (46.4)	
**Cytogenetics**			0.134
** Good**	24 (39.3)	37 (60.7)	
** Intermediate**	28 (58.3)	20 (41.7)	
** Poor**	21 (52.4)	20 (48.8)	
**Hemoglobin, g/dL**			0.730
** < 8.0**	54 (45.0)	66 (55.0)	
** ≥ 8.0**	59 (47.2)	66 (52.8)	
**Platelet, 10**^3^**/uL**			0.566
** < 50**	46 (48.4)	49 (51.6)	
** ≥ 50**	67 (44.7)	83 (55.3)	
**IPSS risk, n (%)**			0.050
** Lower risk**	60 (40.8)	87 (59.2)	
** Higher risk**	53 (53.5)	46 (46.5)	
**WPSS risk, n (%)**			0.005
** Lower risk**	41 (36.3)	72 (63.7)	
** Higher risk**	72 (54.1)	61 (45.9)	
**IPSS-R risk, n (%)**			0.121
** Lower risk**	35 (44.3)	44 (42.6)	
** Higher risk**	34 (57.6)	25 (42.4)	
**HMA treatment**			0.862
** Azacitidine**	81 (46.3)	94 (53.7)	
** Decitabine**	32 (45.1)	39 (54.9)	
**Number of HMA cycles**			0.112
** < 7 cycles**	48 (40.7)	70 (59.3)	
** ≥ 7 cycles**	65 (50.8)	63 (49.2)	
**Response**			0.527
** < PR**	53 (43.8)	68 (56.2)	
** ≥ PR**	49 (48.0)	53 (52.0)	
**Salvage therapy**			0.006
** Chemotherapy**	40 (62.5)	24 (37.5)	
** No or supportive care**	50 (38.2)	81 (61.8)	
** Unknown**	23 (45.1)	28 (54.9)	

HMA: hypomethylating agents, ECOG: Eastern Cooperative Oncology Group performance status, BM: bone marrow, IPSS: international prognostic scoring system, WPSS: WHO adapted prognostic scoring system, IPSS-R: revised international prognostic scoring system, PR: partial response.

The differences in survival between lower and higher risk MDS are as follows. In lower risk MDS, 3 years PFS of treatment failure group and discontinuation group are 6.1% and 54.5%, respectively (*P* < 0.001) and 3 years OS of treatment failure group and discontinuation group are 36.2% and 72.6%, (*P* < 0.001) respectively. In higher risk MDS, 3 years PFS of treatment failure group and discontinuation group are 7.8% and 19.5%, respectively (p = 0.001) and 3 years OS of treatment failure group and discontinuation group are 19.3% and 20.1%, (p = 0.994) respectively.

**Figure 2:**
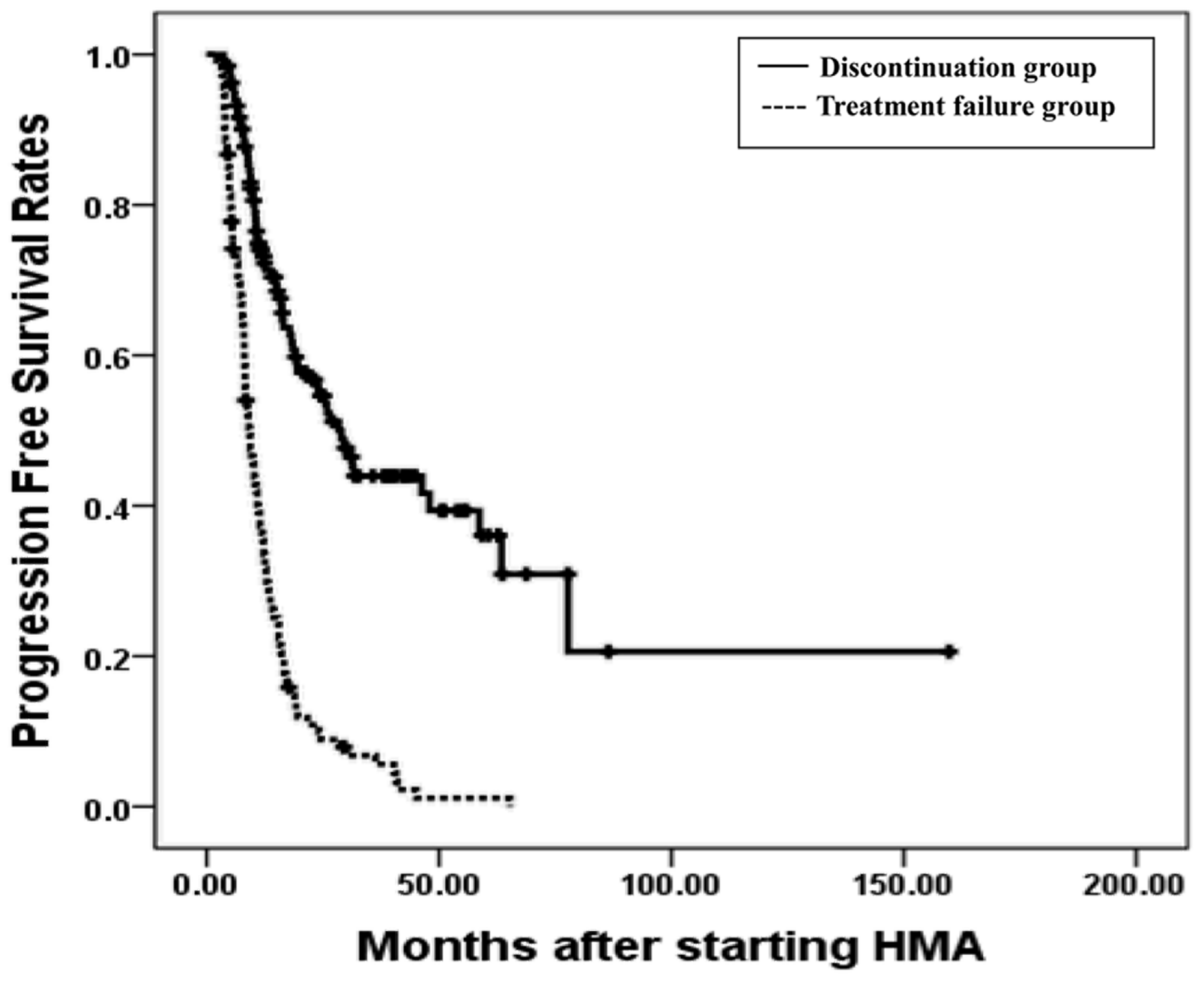

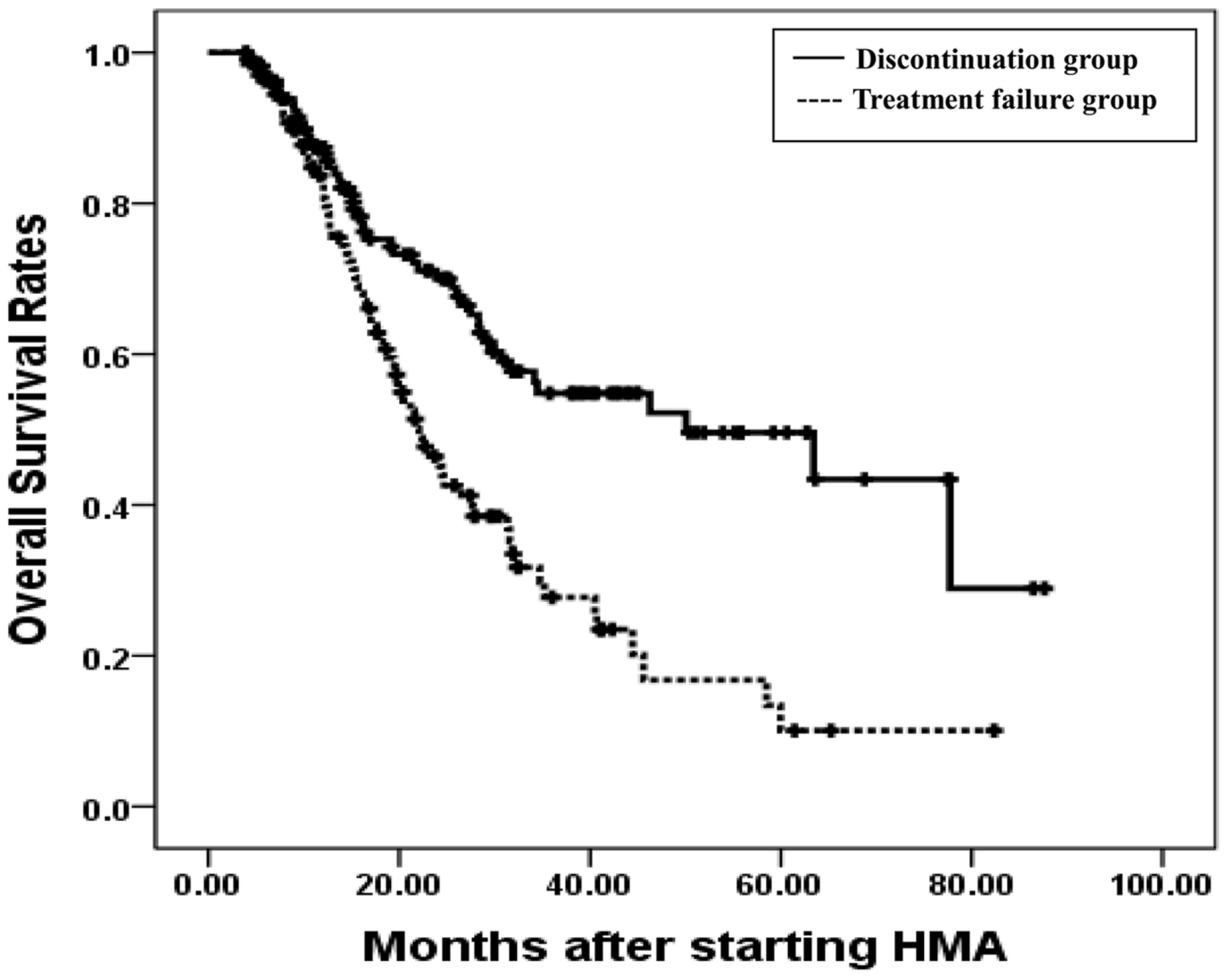
The 3 years progression free survival rates (PFS) and overall survival rates(OS) The 3 years progression free survival rates (PFS) in treatment failure and discontinuation group were 6.8% and 44.0%, *P* < 0.001, respectively **(a)**, and the 3 years overall survival rates (OS) in two groups were 27.8% and 54.8%, *P* < 0.001, respectively **(b)**.

In a univariate analysis, bone marrow myeloblasts less than 5%, good cytogenetics, lower risk by IPSS, WPSS, and IPSS-R, median number of HMA cycles greater than 7, and discontinuation of HMA were significant predictors of a longer PFS (Table 2). An age less than 65 years, median number of HMA cycles more than 7, bone marrow myeloblasts less than 5%, good cytogenetics, lower risk by IPSS, WPSS, and IPSS-R, and discontinuation of HMA were significant predictors of longer OS in all patients as well (Table 2). In a multivariate analysis, intermediate cytogenetics was an independent poor risk factor for PFS (Hazard ratio (HR): 0.617, *P* = 0.043) and discontinuation of HMA was an independent good risk factor for PFS (HR: 5.864, *P* < 0.001). An age greater than 65 years, poor cytogenetics, a higher risk by WPSS (HR: 0.579, *P* = 0.041, HR: 0.176, *P* < 0.001, and HR: 0.290, *P* = 0.011, Table 2), median number of HMA cycles more than 7, and discontinuation of HMA were also independent good risk factors for OS (HR: 2.301, *P* < 0.001, and HR: 2.212, *P* = 0.006, Table 2). Of note, the median PFS was 9.2 months (range 7.7 – 10.7 months) vs 28.9 months (range 22.6 – 35.2) in the treatment failure and discontinuation groups, respectively (*P* < 0.001, Figure 2A). The median OS was 22.1 months (range 18.2 – 26.0 months) vs 50.1 months (range 23.9 – 76.3 months) in the two groups, respectively (*P* < 0.001, Figure 2B).

**Table 2:** Univariate and multivariate analysis for survival in all patients (n=246)

Value	Univariate				Multivariate					
	3yrs PFS		3yrs OS		3yrs PFS			3yrs OS		
		*P*-value		*P*-value	HR	95% C.I	*P*-value	HR	95% C.I	*P*-value
**Age, years**										
** < 65**	^28.7^	0.463	^51.6^	0.016						
** ≥ 65**	24.2		36.0					0.579	0.343∼0.978	0.041
**BM blast, %**										
** < 5**	^37.8^	<0.001	^60.1^	<0.001						
** ≥ 5**	11.4		24.2		0.826	0.428∼1.594	0.568	0.475	0.214∼1.053	0.067
**Cytogenetics**										
** Good**	^43.8^	<0.001	^49.6^	<0.001						
** Intermediate**	^9.1^		^28.5^		0.617	0.328∼1.161	0.043	0.304	0.146∼0.634	0.001
** Poor**	11.1		17.3		1.268	0.694∼2.318	0.134	0.176	0.080∼0.388	<0.001
**Platelet, 10**^3^**/uL**										
** < 50**	^21.1^	0.397	^34.1^	0.051						
** ≥ 50**	29.1		48.0					1.376	0.809∼2.342	0.239
**IPSS risk, n (%)**										
** Lower risk**	^34.3^	<0.001	^57.3^	<0.001						
** Higher risk**	12.6		19.6		1.298	0.697∼2.417	0.411	1.619	0.863∼3.037	0.133
**WPSS risk, n (%)**										
** Lower risk**	^44.7^	<0.001	^65.3^	<0.001						
** Higher risk**	9.2		22.2		0.509	0.245∼1.058	0.070	0.290	0.112∼0.752	0.011
**IPSS-R risk, n (%)**										
** Lower risk**	^36.2^	<0.001	^49.3^	<0.001						
** Higher risk**	4.6		8.5		0.637	0.369∼1.099	0.105	1.425	0.722∼2.812	0.308
**HMA treatment**										
** < 7 cycles**	^23.3^	0.014	^34.7^	0.002						
** ≥ 7 cycles**	28.2		49.1		0.860	0.560∼1.320	0.491	2.301	1.401∼3.778	0.001
**HMA group**										
** Treatment failure**	^6.8^	<0.001	^27.8^	<0.001						
** Discontinuation**	44.0		54.8		5.864	3.589∼9.579	< 0.001	2.212	1.255∼3.897	0.006

3 yrs PFS: 3 years progression free survival rates; 3 yrs OS: 3 years overall survival rates; HR: hazard ratio; BM: bone marrow; IPSS: international prognostic scoring system; WPSS: WHO adapted prognostic scoring system; IPSS-R: revised international prognostic scoring system; HMA: hypomethylating agents.

### Factors associated with survival in the discontinuation group

However, further analyses proceeded to estimate predictive factors for a longer PFS and OS in the discontinuation group (*n* = 133). In a univariate analysis, some factors, such as bone marrow myeloblasts < 5%, good cytogenetics, lower risk by IPSS, WPSS, and IPSS-R, and median number of HMA cycles more than 7, were significant predictive factors for a longer PFS and OS in the discontinuation group (Table 3). In a multivariate analysis, a higher risk by WPSS was an independent poor risk factor for PFS (HR: 0.244, *P* = 0.026) and OS (HR: 0.146, *P* = 0.014). A median number of HMA cycles more than 7 was an independent good risk factor for OS (HR: 4.043, *P* = 0.005) in only the discontinuation group (Table 3).

**Table 3:** Univariate and multivariate analyses for survival in discontinuation group (n=133)

Value	Univariate				Multivariate					
	3yrs PFS		3yrs OS		3yrs PFS			3yrs OS		
		*P*-value		*P*-value	HR	95% C.I	*P*-value	HR	95% C.I	*P*-value
**BM blast, %**										
** < 5**	^56.8^	0.002	^69.5^	< 0.001						
** ≥ 5**	20.9		30.8		1.762	0.569∼5.458	0.326	1.098	0.321∼3.756	0.882
**Cytogenetics**										
** Good**	^69.2^	0.001	^67.3^	0.044						
** Intermediate**	^26.5^		^38.9^		0.594	0.146∼2.419	0.467	0.488	0.097∼2.458	0.385
** Poor**	17.7		34.0		1.038	0.276∼3.898	0.956	0.574	0.089∼3.708	0.560
**IPSS risk, n (%)**										
** Lower risk**	^54.5^	0.001	^70.5^	< 0.001						
** Higher risk**	19.5		20.1		1.124	0.407∼3.105	0.822	0.782	0.276∼2.212	0.643
**WPSS risk, n (%)**										
** Lower risk**	^65.4^	< 0.001	^76.0^	< 0.001						
** Higher risk**	15.5		26.4		0.244	0.071∼0.842	0.026	0.146	0.032∼0.679	0.014
**IPSS-R risk, n (%)**										
** Lower risk**	^59.3^	< 0.001	^61.9^	0.032						
** Higher risk**	8.7		18.5		0.405	0.104∼1.570	0.191	1.387	0.232∼8.283	0.720
**HMA treatment**										
** < 7 cycles**	^37.3^	0.044	^46.1^	0.007						
** ≥ 7 cycles**	51.0		64.7		0.967	0.436∼2.142	0.934	4.043	1.525∼10.720	0.005

3 yrs PFS: 3 years progression free survival rates; 3 yrs OS: 3 years overall survival rates; HR: hazard ratio; BM: bone marrow; IPSS: international prognostic scoring system; WPSS: WHO adapted prognostic scoring system; IPSS-R: revised international prognostic scoring system; HMA: hypomethylating agents.

## DISCUSSION

In this retrospective analysis, patients who discontinued HMA without disease progression showed a superior PFS and OS than those who failed HMA treatment. Comparing the two groups, there were more patients with bone marrow myeloblasts < 5% and a lower risk by IPSS and WPSS in the discontinuation group than in the treatment failure group (Table 1). These differences in baseline characteristics might affect PFS and OS in both groups. So, further evaluation proceeded to confirm whether discontinuation of HMA treatment affects differences of survival between the two groups or not. In a multivariate analysis, independent prognostic factors for OS were age, bone marrow myeloblast percent, cytogenetic risk, number of HMA cycles, and a prognostic risk model, which have already been documented as factors in previous studies [14–16]. Especially, discontinuation or failure of HMA treatment was found as an independent prognostic factor for PFS and OS in a multivariate analysis. However, more patients with lower risk MDS were included in the discontinuation group than the treatment failure group, which was likely due to physician discretion. Until now, some previous studies reported that long term use of HMA improved survival in patients with higher risk MDS [21, 22]. The recommendations were to continue azacitidine until progression in patients achieving at least stable disease with hematologic improvement [23]. But, there are no data supporting the interruption of azacitidine before progression in responding patients. So, in real clinical practice, physicians tend to continue HMA treatment in higher risk MDS; but, patients with lower risk MDS often stopped more frequently, especially as a result of other causes such as toxicities, poor economics, comorbidities, compliance, etc., without disease progression. Actually, patients with lower risk MDS were more prominent in the discontinuation group than in the treatment failure group in our study (Table 1).

However, Cabrero M et al. presented a series of 16 patients with higher-risk MDS (*n* = 5; 31%) or AML (*n* = 11; 69%) who achieved PR (*n* = 1) or CR (*n* = 15) and stopped HMA therapy [19]. They suggested that patients who received 12 cycles of therapy or more had a significantly better OS (median: 20 months [95% CI: 12–27]) than those who received fewer than 12 cycles (median: 4 months [95% CI: 1–8]) (*P* = 0.043). Poor-risk cytogenetics were also associated with a lower 1-year OS (33% versus 69%; *P* = 0.046). Jabbour EJ et al, reported about outcomes of HMA failure. After a median follow-up of 21 months from decitabine failure, 13 (15%) patients remained alive; the median survival was 4.3 months [16]. And from a report on behalf of the MDS Clinical Research Consortium, the median transformation-free survival and OS after HMA failure were 15 months and 17 months, respectively. The estimated 12-month survival rates were 90%, 77%, 37% and 39%, respectively, for patients with low-risk, intermediate-1-risk, intermediate-2-risk, and high-risk disease by the IPSS. Baseline neutropenia, intermediate-risk and poor-risk baseline karyotype, and lack of response to HMA were found to be associated with a higher risk of disease progression [15]. In this study, lower risk by WPSS was an independent risk factor for a longer PFS, and lower risk by WPSS and a median number of HMA cycles greater than 7 were independent risk factors for a longer OS only in the discontinuation group (Table 3). According to our results, discontinuation of HMA can be considered carefully in patients who were shown as lower risk by prognostic models and who sustained a good response with long term HMA. However, there were some limitations to this study. As our data were collected retrospectively, analyzed raw data were often not sufficient or fully exact. The forty four and sixty four patients categorized by IPSS and WPSS, respectively, could not be analyzed for survival by IPSS-R because of insufficient cytogenetic data. Additionally, twenty two patients had insufficient medical data because of follow up loss.

In conclusion, patients without disease progression who discontinued HMA showed a prolonged survival compared to those who failed HMA treatment in clinical practice. In particular, patients with a lower risk by WPSS and long term use of HMA for at least seven cycles may have a prolonged PFS and OS by discontinuing HMA. However, further studies are needed to determine which patients can be considered for discontinuation of HMA and how many cycles of HMA treatment may be optimal for longer survival in true clinical practice.

## MATERIALS AND METHODS

### Patients and study design

A total of 335 patients who satisfied the following criteria were enrolled at fourteen university hospitals in South Korea between January 2001 and October 2013. The medical records of this study were collected retrospectively from a nationwide registry, which was performed with the AML/MDS working party in South Korea. All included patients had been newly diagnosed with MDS and were treated with HMAs, such as azacitidine or decitabine, as front line therapy continuously for at least 4 cycles. Patients were excluded from this study if they had received allogenic stem cell transplantation, showed early death, or ceased treatment without a response evaluation before at least 4 cycles of HMA. Patients with unsatisfactory raw medical recording data were also excluded. The study flow chart is shown in Figure 1. All patients were treated with decitabine 20 mg/m^2^ for 5 days every 4 weeks or azacitidine 75 mg/m^2^ for 7 days every 4 weeks.

Patients were divided into two groups: treatment failure, which are those who discontinued HMA treatment due to disease progression, and the discontinuation group, those who stopped HMA treatment because of other causes such as toxicity, poor economics, comorbidities, compliance, etc. The toxicity was determined by the investigator's judgment at each institution when further treatment is impossible because if hematologic or non-hematologic toxicity is at least National Cancer Institute (NCI) Common Terminology Criteria for Adverse Events (CTCAE) grade 3 to 4. All patients were allowed salvage therapy at the discretion of a clinician. Cytogenetic analysis from bone marrow samples of patients was carried out according to standard procedures. Where possible, 20 or more metaphases from each patient were analyzed in order to demonstrate the clonal nature of the aberrations. The cytogenetic risk, which was determined by conventional cytogenetics, was categorized as good, intermediate, or high risk. Good risk included normal cytogenetics and diploid/loss of chromosome Y, deletion in 5q only, and deletion in 20q only. High risk factors included complex abnormalities that involved ≥ 3 changes or a chromosome 7 abnormality, either alone or with other abnormalities. Intermediate risk was defined as not meet the conditions of either low or high risk. Treatment response was assessed with the 2006 modified international working group response criteria. Besides induction of CR and partial response (PR), achievement of hematologic improvement (HI) according to the International Working Group (IWG) 2006 criteria, i.e., improvement in cytopenias (mainly anemia and/or thrombocytopenia), should be considered indicative of response to treatment because it has been shown to be associated with a prolongation of survival [20]. Responses to HMA and subsequent therapies were coded according to the 2006 IWG Criteria for response assessment in patients with MDS. Response evaluation was performed at least every 4 cycles. BM assessment was performed for patients with BM blasts greater than 5% after 3-4 cycles of treatment. Disease progression was defined as at least a 50% decrease from the maximum response in granulocytes or platelets, a reduction in hemoglobin by more than 2 g/dL, or change to transfusion-dependence. Treatment failure was defined as no response after at least 4 cycles of therapy, loss of response, progression to higher-risk MDS categories, or transformation to acute myeloid leukemia (AML).

### Statistical analysis

PFS was defined as the duration from the start date of HMA therapy to the date of disease progression, relapse, or death from any causes. OS was defined as the duration from the start date of HMA therapy to the date of death from any cause or the final follow-up date. Survival probabilities were calculated using the Kaplan-Meier method, assessed from starting HMA therapy, and compared using the log-rank test. Univariate and multivariate analyses were performed to identify potential prognostic factors associated with PFS and OS. In this study, previous reported prognostic factors such as poor performance, older age, thrombocytopenia, anemia, increased bone marrow blasts, leukocytosis, chromosome 7 or complex (≥ 3) abnormalities, prior transfusions, and risk models, including the international prognostic scoring system (IPSS), WHO adapted prognostic scoring system (WPSS), and revised IPSS (IPSS-R) risk model, were analyzed; treatment failure, discontinuation of HMA, and number of HMA cycles were additionally estimated in this study. The Cox proportional hazard regression analysis was used for PFS and OS. Information about baseline medical status and treatment modalities were collected from the medical records. Approval for these studies was obtained from the Institutional Review Board.

## Abbreviations

HMA: hypomethylating agent
PFS: progression free survival
OS: overall survival
MDS: myelodysplastic syndrome
AML: acute myeloid leukemia
CR: complete response
PR: partial response
HI: hematologic improvement
IWG: International Working group
IPSS: international prognostic scoring system
WPSS: WHO adapted prognostic scoring system
IPSS-R: revised IPSS
SD: stable disease
HR: hazard ratio
